# Assessment of public health interventions for decision support: methods & processes of the evaluation of the Austrian screening programme for pregnant women & children

**DOI:** 10.1007/s10354-019-0690-2

**Published:** 2019-03-12

**Authors:** Inanna Reinsperger, Katharina Rosian, Roman Winkler

**Affiliations:** Ludwig Boltzmann Institute for Health Technology Assessment, Garnisongasse 7/20, 1090 Vienna, Austria

**Keywords:** Evidence-based guidelines, Screening, Pregnancy, Children, Appraisal process, Evidenzbasierte Leitlinien, Screening, Schwangerschaft, Kindheit, Appraisal-Prozess

## Abstract

**Background:**

In 2010, the Austrian Ministry of Health (MoH) commissioned the Ludwig Boltzmann Institute for Health Technology Assessment (LBI-HTA) to prepare decision support for the reorientation of the national screening programme for pregnant women and children (“Mutter-Kind-Pass”). This is one of several reports aimed at giving an overview of recommended screening measures.

**Methods:**

By conducting a guideline synopsis, we summarised screening recommendations from guidelines of high methodological quality. To facilitate contextualisation of the recommendations, the MoH initiated a transparent appraisal process.

**Results:**

We identified 101 guidelines dealing with screenings during pregnancy and 75 guidelines providing screening recommendations for children. From 2014 to 2018, an interdisciplinary and multi-professional expert group discussed these results and formulated recommendations for a new Austrian screening programme.

**Conclusion:**

Following the multi-step process of Health Technology Assessment (assessment–appraisal–decision), this methodological approach bridges evidence and national expert knowledge and represents a novel path towards a new screening programme in Austria.

## Zusammenfassung

**Grundlagen:**

Das Ludwig Boltzmann Institut für Health Technology Assessment (LBI-HTA) wurde im Jahr 2010 vom österreichischen Gesundheitsministerium beauftragt, eine Entscheidungsunterstützung für eine Neuorientierung der Eltern-Kind-Vorsorge („Mutter-Kind-Pass“) zu erarbeiten. Einer der zahlreichen Forschungsberichte hatte das Ziel, eine Übersicht zu empfohlenen Screening-Maßnahmen für die Schwangerschaft und die frühe Kindheit zu erarbeiten.

**Methodik:**

Screening-Empfehlungen aus Leitlinien hoher methodologischer Qualität wurden mittels Leitliniensynopse zusammengefasst. Zur Kontextualisierung dieser Empfehlungen initiierte das Gesundheitsministerium einen transparenten Appraisal-Prozess.

**Ergebnisse:**

Es wurden 101 Leitlinien zu Screenings während Schwangerschaft und Wochenbett sowie 75 Leitlinien zu Screenings für Kinder identifiziert. Zwischen 2014 und 2018 diskutierte eine interdisziplinäre und multiprofessionelle Arbeitsgruppe diese Ergebnisse und formulierte Empfehlungen für ein neues Screening-Programm.

**Schlussfolgerung:**

Dem Health Technology Assessment-Zyklus „assessment – appraisal – decision“ folgend, verbindet dieser methodologische Ansatz die Evidenz mit nationaler Expertise und stellt einen innovativen Prozess zur Weiterentwicklung des Mutter-Kind-Passes in Österreich dar.

## Introduction

Complex health interventions (such as screening programmes) are defined by multiple interventions carried out by manifold actors in different settings. This requires the consideration of a spectrum of outcomes and processes. In order to understand the complexity behind these interventions, research and decision support needs to transcend the mere question of ‘what works?’ In fact, additional factors need to be considered. This involves questions focusing on target groups (e. g., general vs. specific population), their attitudes towards an intervention (e. g., in favour of or against specific health services), the health care setting (e. g., inpatient or outpatient context), the relevant health care providers (e. g., medical doctors, nurses or other health care professionals), etc. [1]. Hence, the involvement of health experts, their deliberate selection, and the consideration of their expertise is decisive for answering the above questions adequately.

In 1974, Austria introduced a nationwide accessible screening programme (‘Mutter-Kind-Pass’) for pregnant women and their children up to the age of 6 years. Since then, the range of screening tests has enlarged, but a comprehensive evaluation of the programme is still lacking. In 2010, the Austrian Federal Ministry of Health (MoH) commissioned the Ludwig Boltzmann Institute for Health Technology Assessment (LBI-HTA) to provide decision support for the reorientation of the mother–child screening programme that aimed to address socio-medical as well as clinical health risks. For this purpose, our research was based on the screening definition of the World Health Organization (WHO), which describes screening as *‘the presumptive identification of unrecognised disease in an apparently healthy, asymptomatic population by means of tests, examinations or other procedures that can be applied rapidly and easily to the target population’* [2]. During the past years, more than ten LBI-HTA reports have dealt with different research questions concerning screening and prevention measures during pregnancy and early childhood (e. g., epidemiology of health threats and risk factors, scope of international screening programmes, effectiveness of interventions to counteract preterm birth, financing structures in Austria, organisation of health visiting services) [3] using various methods (e. g., overview of systematic reviews, budget impact analysis, expert survey). One major part of the series of inputs to the re-orientation of the Austrian Mutter-Kind-Pass was a systematic overview of guideline recommendations on antenatal and postnatal screenings [4]. The LBI-HTA project team chose to conduct a ‘guideline synopsis’ given the project aim: to provide a broad overview of a wide range of different screening topics that are potentially relevant during pregnancy and early childhood.

This methodology is a novel approach to dealing with multiple guidelines. Several other institutions regularly conduct and/or publish such overviews of guideline recommendations, mostly called ‘guideline synopses’. Guideline-developing institutions, such as the Association of the Scientific Medical Societies in Germany (AWMF), give guidance on how to identify relevant national and international guidelines and how to use them for the development of a new one. This approach aims at limiting efforts, particularly regarding the search for relevant literature for the guideline [5]. The ADAPTE Collaboration published a toolkit in 2009 providing a systematic approach to adapting guidelines for use in a different cultural and organisational setting [6]. Apart from the LBI-HTA, there are other research institutions conducting guideline synopses: the German Institute for Quality and Efficiency in Health Care (IQWiG) prepared guideline synopses as a basis for disease management programmes (DMP) in Germany [7]; the Department for Evidence-Based Medicine and Clinical Epidemiology of the Danube University Krems published a guideline synopsis on screening for adolescents aged 16–18 years [8]; the Institute of General Practice and Evidence-Based Health Services Research of the Medical University of Graz conducted, for example, a guideline synopsis on the therapy and prevention of chronic obstructive pulmonary disease (COPD) [9].

Guidelines *are ‘systematically developed statements reflecting the current state of knowledge and meant to support doctors and patients in making decisions concerning appropriate care for specific health problems*’ [5]. High-quality evidence-based guidelines are developed based on a systematic literature search and analysis of relevant articles. The recommendations are explicitly linked to the underlying evidence and the guidelines are regularly checked to ensure currentness. Evidence-based guidelines summarise the currently available evidence and assess the balance between the benefits and potential harms of interventions. Additionally, and in contrast to other sources of aggregated evidence such as systematic reviews, they give explicit practice recommendations. That makes them a valuable source of information for guideline synopsis, not only for clinicians and other health professionals, but also for policy decision-makers [10].

In this article, we describe the methods of synthesising recommendations from guidelines dealing with preventive care for pregnant women and children aged 0–6 years. Additionally, we show how the results of the guideline synopsis supported the subsequent appraisal process.

## Methods and process

First, we provide a detailed description of the methodological steps for preparation of a guideline synopsis. Secondly, we describe how the guideline synopsis was used for a novel appraisal process initiated by the MoH to facilitate an appraisal of the guidelines and the formulation of recommendations for a new screening programme for pregnancy and early childhood in Austria.

### Guideline synopsis

Generally, conducting a guideline synopsis necessitates taking into account the following constituent steps: definition of inclusion criteria, literature search, selection of guidelines and assessment of guideline quality, data extraction and summary of guideline recommendations.

The synopsis process started with the definition of inclusion criteria for relevant guidelines, involving

- the target population (e. g., pregnant women in general, children from 0 to 6 years old),
- the intervention (e. g., screening, diagnosis, therapy and management),
- the outcomes of interest (screening recommendations),
- the study design (only evidence-based guidelines) and publication period.

To ensure that guidelines include the most recent evidence, they need to be assessed regularly for their validity and updated if necessary. Most guideline institutions choose a period of 3–5 years before they perform a validity check or a guideline update [5, 11, 12]. Table 1 summarises the inclusion criteria that we applied for our guideline synopsis.

**Table 1:** Inclusion criteria

Population	Pregnant women and children from 0 to 6 years
Intervention	Screening (single screening tests, screening programmes, identification of risk factors, etc.)
Outcomes	Screening recommendations Grade of recommendation Timing of screening Screening methods Therapy options Potential screening harms
Study design	Evidence-based guidelines from Western industrialised countries
Publication period	Published or reaffirmed during the past 5 years

The second step was the literature search. We searched for guidelines in two guideline databases: the National Guideline Clearinghouse (NGC) and the Guideline International Network (G-I-N). Additionally, we identified relevant guidelines through a hand search on websites of guideline-developing institutions, for example, the U.S. Preventive Services Task Force (USPSTF), the National Institute for Health and Care Excellence (NICE), the Canadian Task Force on Preventive Health Care (CTFPHC) or the Association of the Scientific Medical Societies in Germany (AWMF, in German). We also searched websites of topic-specific institutions and societies, e. g., the Society of Obstetricians and Gynaecologists of Canada (SOGC) or the Royal College of Obstetricians and Gynaecologists (RCOG).

Consequently, the guideline selection process followed a two-step process: First, we screened the reference titles and excluded guidelines with titles outside the project scope. Second, we assessed the full texts to decide whether they meet the inclusion criteria (see Table 1). We chose this approach because guidelines mostly do not provide abstracts, so the usual initial abstract screening is unfeasible. As we aimed to include only evidence-based guidelines of high quality, guidelines also had to fulfil some methodological quality criteria in order to be finally included in the synopsis: the guidelines had to be based on a systematic literature search and the recommendations had to be explicitly linked to the underlying evidence (mostly by using levels of evidence and/or grades of recommendations). We excluded guidelines that were based only on expert consensus without systematically evaluating and incorporating the current best-available evidence.

After the final inclusion of the guidelines, we extracted the screening recommendations, the grades of recommendation, potential harms linked to particular screenings, the recommended timing of screening, as well as the screening methods from the included guidelines, and prepared a comprehensive report for the Austrian MoH. Further details concerning the methods used for the guideline synopsis can be found in the respective LBI-HTA reports [4, 13, 14].

### Appraisal process

In order to facilitate the appraisal of the prepared guideline synopsis, the Austrian MoH initiated a multi-professional and transparent appraisal process in 2014. The aim of this process was to evaluate the LBI-HTA assessments (in particular the guideline synopsis and the report on the epidemiological data of health threats) in the Austrian context and to formulate recommendations for the development of a new national screening programme for pregnant women and children. The MoH commissioned an interdisciplinary and multi-professional expert working group consisting of obstetricians, paediatricians, general practitioners, midwives, public health experts, social workers, nurses, evidence-based medicine experts and psychologists, as well as experts from the MoH, the Ministry of Family and the Main Association of the Austrian Social Security Institutions. For the meetings of the expert working group, the LBI-HTA project team prepared and presented information from the guideline synopsis, as well as additional background data (e. g., epidemiological data on the respective health threats and threats in question) to the group. The task of the expert group was then to discuss the recommendations from the evidence-based guidelines, add other relevant information from research and/or practice, and specifically include the Austrian context into the discussion. Subsequently, the expert group voted for or against the inclusion of the screening into the new Austrian screening programme. An external moderator guided the discussion and the votes. To structure and focus the discussions, a table with leading questions was used for each potential health threat. These questions referred to the WHO screening criteria published in 1968, as well as to extended and revised versions by other guideline institutions such as the UK National Screening Committee (UK NSC) (see Table 2; [15–17]).

**Table 2:** Criteria for the appraisal of each disease or risk factor

Disease/risk factor/health problem:	+	~	−	?
What do evidence-based guidelines recommend?				
Which grades of recommendation were assigned to the identified recommendations?				
Is the disease/risk factor relevant for Austria (regarding prevalence as well as severity)?				
Does early detection lead to better treatment outcomes?				
Are treatment/intervention options available in Austria?				
Is a screening test available in Austria and is it feasible and accepted by the affected population in this context?				
Do the overall benefits of screening outweigh the potential harms?				
Is the cost/benefit ratio appropriate?				
RECOMMENDATION □ pro screening □ contra screening				

For each criterion listed in Table 2, the expert working group had to decide whether it is met (+), not met (−), contradictory/unclear (~) or if this question cannot be answered (?) (e. g., because of lacking data or evidence). The first two criteria relate to the results of the guideline synopsis. For example, if several guidelines recommend a screening for the respective health problem, the + was chosen; in case of contradictory guideline recommendations the ~ had to be selected. For the subsequent criteria, the Austrian perspective had to be taken into account. The expert working group had to decide, for instance, whether a suitable screening test is available in Austria, whether pregnant women accept this test, whether appropriate treatment options are available, whether the benefits of the screening outweigh potential harms, etc. After the discussion and completion of the appraisal scheme, the voting took place. The voting ratio was noted in the minutes. If more than 75% of the members voted for either screening or no screening, the vote was counted as a consensus. Relevant details such as the target group of the screening (e. g., all children, high-risk pregnant women), the recommended timing of screening (e. g., once during pregnancy, at every childhood examination), the screening tests (e. g., laboratory test, ultrasound, anamnesis, questionnaire) and the implications of a positive result (e. g., further diagnostics, specialist referral, treatment) were also specified and recorded in the minutes.

## Results

### Results of the guideline synopsis

Regarding screening in pregnancy and puerperium, we identified a total of 101 evidence-based guidelines from 12 institutions that fulfilled the inclusion criteria. These guidelines provided recommendations dealing with 48 potential health threats during pregnancy and six health threats during puerperium. Screening topics ranged from various infections (e. g., HIV, rubella) and other diseases of the pregnant woman (e. g., gestational diabetes, anaemia) to foetal anomalies and conditions (e. g., trisomy 21, neural tube defect, foetal growth) and psychosocial issues (e. g., depression, substance misuse or domestic violence) [13]. Regarding screening during early childhood, we included 75 evidence-based guidelines that were developed by ten different institutions. The identified guidelines provided screening recommendations dealing with 45 potential health threats and risk factors during early childhood. These included, for example, overweight/obesity, several mental health problems, dental disease, vision and hearing problems [14].

The provided guideline synopsis gives an overview of screening recommendations from high-quality evidence-based guidelines. These summarise the currently available evidence and assess the balance between benefits and potential harms of interventions. One of the advantages of guidelines is that they not only summarise the evidence (such as systematic reviews), but they also give explicit practice recommendations, for example, for or against a routine screening. Formulation of these recommendations necessitates consideration of (national) contextual factors (such as specific health service structures, epidemiological conditions, etc.). As these contextual factors may differ from one country to another, guideline recommendations cannot just be accepted without further adaptation; an appraisal process is needed to take the national context into account and to formulate own recommendations.

### Results from the appraisal process

Between November 2014 and May 2018, 37 meetings of the expert working group took place. For pregnancy, the expert working group agreed upon 32 recommendations in favour of screening and 27 recommendations against screening. Regarding screening during puerperium, the experts put forward three pro-screening and three contra-screening recommendations. For early childhood, the expert working group recommended screenings for 16 health threats, whereas seven health threats received negative votes (against screening). Furthermore, counselling was recommended for several health topics, either in addition to the screening or when a screening was not recommended. The minutes of the expert working group meetings were published on the MoH website [18, 19], and people and institutions (e. g., health experts, lay people, patients, non-governmental organisations, etc.) who were not involved in the process had the opportunity to submit written comments to the minutes, which were also made publicly available on the website.

### Illustration of the process using the example ‘screening for depression and anxiety disorder’

In the following paragraphs, we will illustrate the process from the assessment to the appraisal and finally to the recommendation, using screening for depression and anxiety disorder in the pregnant woman/mother as an example:

Regarding prenatal and postnatal screening for depression, our guideline synopsis identified seven guidelines that met the inclusion criteria. Of these, five guidelines were in favour of a routine screening for depression [20–24], whereas two institutions recommended no routine screening [25, 26]. The guidelines recommending screening agreed that a screening should be offered at least once during pregnancy and once in the postpartum period. However, there was no consensus on the recommended screening tests. The NICE guideline [20] additionally recommended screening for anxiety disorders. The following table summarises the guideline recommendations providing the decision basis for the appraisal expert group (Table 3).

**Table 3:** Guideline recommendations for depression and anxiety disorder screening

Institution, publication year	Screening recommended (✓) or not recommended (✗)	Grade of recommendation	Recommended timing of screening	Recommended screening test
CTFPHC, 2013 [26]	✗	Weak recommendation	NR	NR
AHMAC, 2012 [23]	✓	B	As early as practical in pregnancy, preferably twice during pregnancy and twice during the first year after birth	EPDS
SIGN, 2012 [24]	✓	D	At minimum on booking in and postnatally (at 4–6 weeks and 3–4 months)	NR
Beyondblue, 2011 [22]	✓	B	At least once, preferably twice, in both the antenatal and postnatal period (ideally 6–12 weeks after birth)	EPDS
VA/DoD, 2009 [21]	✓	B	At the first contact with healthcare services in both the antenatal and postnatal periods (postnatal: at 4–6 weeks and 3–4 months)	NR
NICE, 2007 [20]	✓	NR	At a woman’s first contact with primary care, at her booking visit and postnatally	Two predefined questions
UK NSC, 2011 [25]	✗	NR	NR	NR

*AHMAC* Australian Health Ministers’ Advisory Council*, CTFPHC* Canadian Task Force on Preventive Health Care, *EPDS* Edinburgh Postnatal Depression Scale, *NICE* National Institute for Health and Care Excellence, *NR* not reported, *SIGN* Scottish Intercollegiate Guidelines Network, *UK NSC* UK National Screening Committee, *VA/DoD* Veterans Affairs/Department of Defence

The overall guideline recommendations, as well as additional information such as prevalence, available screening tools and treatment options, were presented to the appraisal expert group. The group assessed all criteria of the appraisal scheme positively (except the grade of recommendation). An important factor was the high prevalence of depression during pregnancy and in the postpartum period: estimates show that around 10–15% of women suffer from postpartum depression in Austria [27]. The appraisal expert group unanimously recommended the inclusion of a depression and anxiety screening into the envisaged new screening programme. However, deciding on the recommended screening test was challenging. The guidelines were not consistent regarding the recommended screening tool and there was a lack of experience with the use of available screening tools in practice in Austria. Hence, the expert working group formulated the following final recommendation: ‘*All pregnant women/mothers should be screened for depression and anxiety disorders using the PHQ-4 (short form of the Patient Health Questionnaire) as early as possible in pregnancy, at 24–28 weeks of pregnancy as well as 6–8 weeks and 3–5 months after birth. The PHQ-4 consists of the first two questions of the depression module (PHQ-2) and the first two questions of the anxiety module (GAD-2; Generalized Anxiety Disorder). If the woman has 3 or more points on the PHQ-2, the EPDS (Edinburgh Postnatal Depression Scale) should be used for further assessment. If the woman has 3 or more points on the GAD-2, the GAD-7 questionnaire should be used.’ *The recommended interventions in the case of a positive screening result include referral of the woman to a specialist/institution that is qualified for the diagnosis, therapy and follow-up of psychiatric disorders during pregnancy and the postnatal period.

## Discussion

For the guideline synopsis, we analysed 101 guidelines from 12 institutions dealing with screenings during pregnancy and puerperium, and 75 guidelines from ten institutions providing recommendations concerning screenings during early childhood. This guideline synopsis was used for an appraisal process that aimed at formulating recommendations for the Austrian screening programme for pregnancy and early childhood, based on the best available evidence and the know-how and experience of relevant national experts. During 37 meetings, a multi-professional and interdisciplinary expert working group discussed the guideline synopsis results regarding their relevance and transferability into the Austrian context. In total, the expert working group formulated 51 pro-screening recommendations and 37 contra-screening recommendations, as well as several counselling recommendations.

In this paper, we described an approach towards the use of evidence-based guidelines for public health decision-making processes. Basically, guidelines consider the best available evidence. Due to specific national factors, guideline development groups may come to different recommendations. This is especially the case when the underlying evidence is contradictory, of low quality or lacking. Since guideline recommendations cannot be transferred directly to another country’s context, they must undergo an appraisal process considering relevant country-specific factors. These include epidemiology, burden of disease, preferences of the affected population, local availability of screening tests, subsequent diagnostic tests and treatments, involved health professionals, already existing national recommendations, potential screening harms, as well as financial resources and organisational structures. For screenings, the UK NSC ‘Criteria for appraising the viability, effectiveness and appropriateness of a screening programme’ [15] are a helpful guidance for the appraisal process.

The use of strict appraisal criteria is of utmost importance given that screening (by definition) addresses healthy populations (such as pregnant women and young children). Hence, appraisal processes should go beyond the mere effectiveness of (screening) interventions and take into account that every screening test can potentially produce harm—these could be false-positive or false-negative test results, unnecessary diagnostic procedures or treatments, anxiety and other psychological harms, etc.—which makes the balancing of benefits and harms even more important.

Many European countries (e. g., Germany, the Netherlands, the UK) have formal appraisal committees that are commissioned to contextualise HTA assessments and formulate recommendations for health policy making [28]. Following the multi-step process of HTA from ‘assessment’ to ‘appraisal’ and to ‘health policy decision-making’, the guideline synopsis marks the first step (‘assessment’). As a second step, the expert working group nationally contextualised the summarised guideline recommendations (‘appraisal’).

We are aware of the following limitations of our project. First, we did not use a quality appraisal tool such as AGREE II (Appraisal of Guidelines for REsearch & Evaluation) to assess the methodological quality of the identified guidelines for our guideline synopsis. AGREE II is an international tool published in 2010 that assesses the methodological rigour and transparency with which a guideline is developed, using 23 items in six domains [29]. Given the limited project resources and the broad range of screening topics we could not apply AGREE II or a similar tool. However, we set strict inclusion criteria assuring high methodological guideline quality (such as outlined in the Methods section). A limitation of the subsequent appraisal process is the long period of time (3.5 years) that was necessary for the monthly meetings of the expert working group. This was due to the broad range of screening topics and the large demand for discussion for some contested issues (e. g., topics where guideline recommendations are contrary to the Austrian practice). The long time period may have led to the necessity of updating some of the recommendations due to newer evidence, and it may have been the reason for changes in the member composition of the expert group over time.

The interdisciplinary and multi-professional composition of the expert working group represents a novelty in Austria. Although the involved multidisciplinary group of experts faced a number of challenges (due to different professional backgrounds), the composition of the group enabled high-level expert discussions and the inclusion of various professional perspectives.

Overall, this methodological approach bridges evidence and national expert knowledge, and represents an entirely novel path for the formulation of parent–child screening recommendations in Austria. Eventually, it is the Austrian MoH that will mainly design the health policy decision-making process dealing with the re-design of prenatal and postnatal screenings in Austria. The appraised recommendations should contribute considerably to this political process.
